# Effect of the Graston Technique and Cupping Therapy on Pain and Functions in Individuals With Medial Tibial Stress Syndrome: A Randomized Clinical Trial

**DOI:** 10.7759/cureus.48246

**Published:** 2023-11-04

**Authors:** Nikita S Deshmukh, Pratik Phansopkar

**Affiliations:** 1 Musculoskeletal Physiotherapy, Ravi Nair Physiotherapy College, Datta Meghe Institute of Higher Education and Research (DU), Wardha, IND

**Keywords:** pain, runners, medial tibial stress syndrome, cupping therapy, graston technique

## Abstract

Background

The discomfort in the mid-shaft and distal end of the tibia caused by shin splints or tibial periostitis also known as medial tibial stress syndrome (MTSS) is frequently induced by participating in sports or other strenuous activities. The two treatment methods used in this study are the Graston technique and cupping therapy; we have compared cupping with the Graston technique.

Method

It was an interventional study at Ravi Nair Physiotherapy College and Acharya Vinoba Bhave Rural Hospital. A total of 46 participants with MTSS were included in the study. The participants were randomly divided into two groups and treated for three weeks with four weekly sessions.

Result

Statistical analysis was done after the completion of sampling. Paired and unpaired t-tests were used. A p-value of <0.05 was considered significant. The result was obtained after comparing the pre and post values of the visual analog scale (VAS), treadmill test, step-up and step-down tests, manual muscle testing (MMT), and range of motion (ROM) of the ankle joint. After three weeks of treatment, pain with a p-value of 0.01 S showed a significant effect, and improved functions were reduced in the cupping and Graston technique groups. When compared, cupping therapy showed better results than the Graston technique.

Conclusion

We saw that the cupping therapy might be better than the Graston technique in reducing pain and improving functions.

## Introduction

In individuals who experience recurrent tension in their lower limbs, bone marrow edema is a common symptom in bone remodeling cases due to excessive stress. When the load exceeds a specific threshold, it can result in clinical signs and symptoms attributed to microscopic fatigue, as outlined in the mechanical model [1]. Conversely, periostitis is the inflammation resulting from muscle fiber rupture at the junction between muscle and bone. It presents a significant injury risk, with a prevalence of 9.5% and an incidence rate ranging from 13.6% to 20.2%, especially among long-distance runners [2].

The pain associated with medial tibial stress syndrome (MTSS) can be traced back to the involvement of the soleus muscle fibers based on the traction theory. This is due to its insertion point located approximately 4 inches proximal to the medial malleolus and the origin of the tibialis posterior muscle, which is situated 7.7 cm proximal. One of the key clinical indicators used to diagnose MTSS is foot pronation, which plays a pivotal role in its assessment [3].

Research studies consistently indicate that among recreational runners, MTSS is the most prevalent condition [4]. In mild cases, pain is noticeable during activity, whereas in severe instances, discomfort persists even at rest. Prolonged muscle overuse and fatigue can result in weakness in the posteromedial region [5]. Shin splints can be categorized into two types: anterior leg compartment dysfunction or issues with surrounding structures and posterior leg compartment dysfunction or problems affecting surrounding structures. Soreness in the lower two-thirds of the leg often hampers exercise [5].

Furthermore, MTSS can be attributed to traction periostitis of the soleus or flexor digitorum longus muscle origin, in conjunction with increased heel eversion as contributing factors [6,7]. It is noteworthy that ankle dorsiflexion below 20-30% during closed-chain movements can harm normal locomotion and lead to compensatory gait patterns [8]. Most research points to shin splints as recreational runners' most frequently diagnosed or second most diagnosed condition. In mild cases, discomfort arises during physical activity, while in severe cases, pain is prevalent even during rest [5].

Patients suffering from MTSS typically undergo a treatment regimen that includes gradual jogging, strengthening exercises, and stretching routines primarily focused on the calf muscles. These therapeutic interventions aim to improve the condition and facilitate recovery by incrementally increasing activity levels, enhancing the strength of the affected muscles, and improving flexibility to reduce stress on the tibia and its surrounding structures [9].

The Graston technique stands as a valuable method for enhancing physical condition in the realm of sports. It utilizes specialized instruments to modify the structure and composition of soft tissues, thus enhancing fascia mobility and addressing tissue adhesions. This technique proves beneficial in treating and rehabilitating athletes and non-athletes dealing with repetitive and cumulative injuries. The Graston technique effectively targets problematic areas and fosters tissue healing. It restores optimal function, making it a valuable strategy for managing musculoskeletal conditions and enhancing overall performance in sports and everyday activities [10]. Its core aim is to improve tissue function, alleviate pain, and enhance overall patient mobility, establishing its significance in physical therapy and sports medicine [11].

Cupping, a traditional Chinese medicinal practice that has gained recognition in various regions, including Northern Europe (Scandinavia) and Asia, most notably in China, offers various therapeutic approaches to address specific health concerns. These approaches promote blood circulation, alleviate pain, reduce muscle tension, and cater to diverse health conditions. It is vital to emphasize that cupping therapy should only be administered by trained professionals and customized based on individual health needs and requirements [12].

Cupping is a therapeutic technique that involves the application of negative pressure to the skin over areas experiencing discomfort. This negative pressure is created by applying cups to the skin, inducing suction, and drawing the skin and underlying tissues upward [13]. This study aims to determine the effectiveness of cupping therapy in improving function and reducing pain in cases of MTSS and to compare it with the Graston technique to identify the superior approach.

## Materials and methods

Study design and setting

This experimental trial took place at two distinct healthcare facilities, the Musculoskeletal Outpatient Department (OPD) of Acharya Vinoba Bhave Rural Hospital and the Musculoskeletal Department of Ravi Nair Physiotherapy College, both affiliated with the Datta Meghe Institute of Higher Education and Research (DU), situated in Sawangi (Meghe), Wardha.

Participant selection

A total of 46 individuals diagnosed with MTSS were included in the study using a random sampling method. The participants were evenly divided into two groups, each consisting of 23 individuals, and the allocation to these groups was carried out through the envelope method.

Inclusion and exclusion criteria

The inclusion criteria for participants encompassed recreational runners experiencing symptoms for more than two weeks. This criterion applied to both males and females, aged between 20 and 30 years, with pain localized to the anterior and posteromedial sides of the shin and local tenderness graded as I or II upon palpation over these areas. The diagnosis of MTSS was further confirmed through a physical test, which involved identifying palpable pain across 5 cm or greater and the absence of unique symptoms.

On the other hand, the exclusion criteria were applied to individuals with fractures in the lower limb, a history of surgical procedures around the knee and ankle, previous bone pathologies around the knee and ankle, and underlying deformities, such as genu varum or valgum deformity or hyperextended knee. The criteria also excluded individuals with a history of acute trauma, neurological disease, or other systemic illnesses, including metabolic, metastatic, or infective disorders.

Treatment procedure

After screening participants based on the inclusion and exclusion criteria, those who met the requirements and expressed their willingness to participate were included in the study. Subsequently, these participants were divided into two distinct groups. Group A received treatment using the Graston technique, while Group B received cupping therapy. Both groups underwent a conventional physiotherapy protocol, including 20 minutes of stretching and strengthening exercises. The treatment was administered by a qualified physiotherapist over three weeks, involving four sessions each week.

Outcome measures

Before the commencement of the treatment and upon its completion, several outcome measures were assessed. These included the visual analog scale (VAS) [14], treadmill test [15,16], step-up and step-down tests [17], range of motion (ROM) [18], and manual muscle testing (MMT) [19]. These assessments were conducted to gauge the effectiveness and impact of the respective treatment approaches on the participants' conditions.

Statistical analysis

Statistical analysis was performed using descriptive and inferential statistics, including Student's paired and unpaired t-tests and the software IBM SPSS Statistics for Windows, Version 27.0 (Released 2020; IBM Corp., Armonk, New York, United States) and GraphPad Prism, Version 7.0 (GraphPad Software, San Diego, California, United States), with a p-value of less than 0.05 assumed to be the significance level. Paired t-test was used to compare pre data with post data within the group, while an unpaired t-test was used to compare the post mean between the groups in age-wise distribution.

Ethical considerations

The study was conducted with the requisite approvals from the Institutional Ethics Committee of Datta Meghe Institute of Higher Education and Research (DMIHER, DU) in Sawangi (Meghe), Wardha (DMIMS (DU)/IEC/2022/798). It was registered with the Clinical Trial Registration of India (CTRI/2022/06/043090). Informed consent was diligently obtained from each participant, ensuring their complete understanding of the research protocol. The research protocol was formally published in the Journal of Medical Pharmaceutical and Allied Sciences, under the DOI: 10.55522/jmpas.V11I4.1311.

## Results

**Figure 1:**
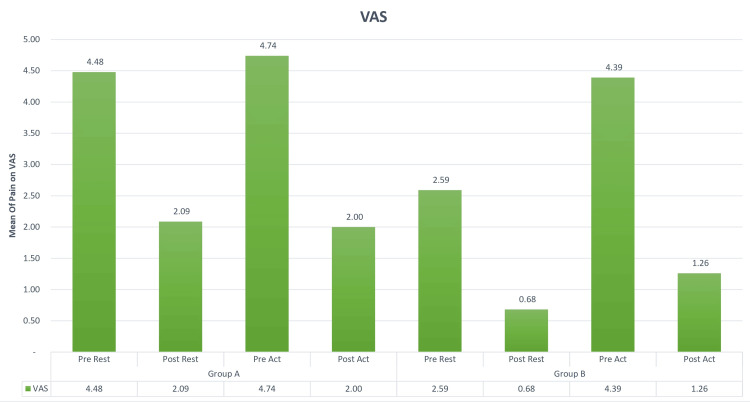
Comparisons of VAS in Groups A and B in the pre and post test. The average VAS score for Group A prior to rest was 4.48, and after rest, it dropped to 2.09. During the pre-activity phase, the mean VAS score was 4.74, and after the activity, it decreased to 2.00. As for Group B, the mean VAS score before rest stood at 2.59, and after rest, it decreased to 0.68. During the pre-activity phase, the mean VAS score was 4.39, and after the activity, it was 1.26 VAS: visual analog scale

**Figure 2:**
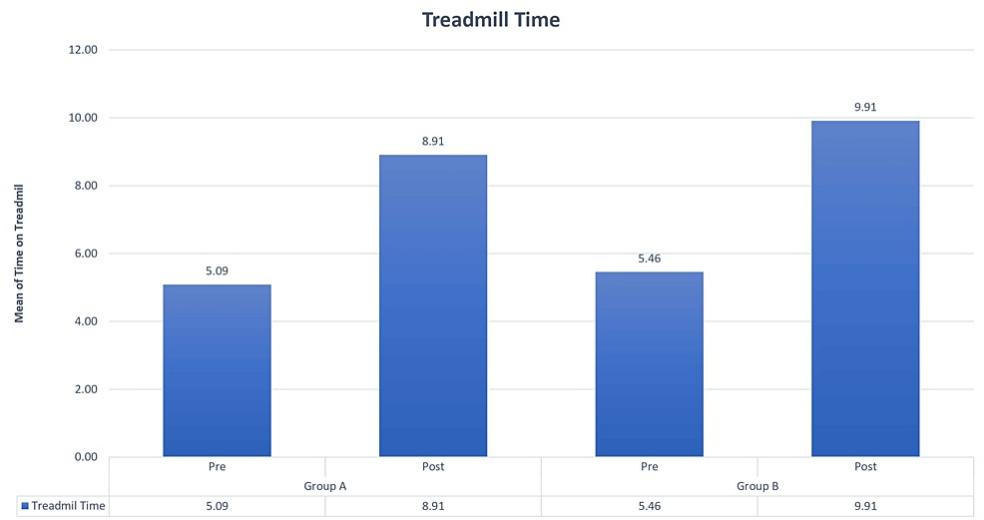
Comparisons of treadmill time in Groups A and B in the pre and post test. During the pre test, the average treadmill time for Group A was 5.09, and during the post test, it increased to 8.91. As for Group B, the mean pre-test treadmill time was 5.46, and it rose to 9.91 in the post test

**Figure 3:**
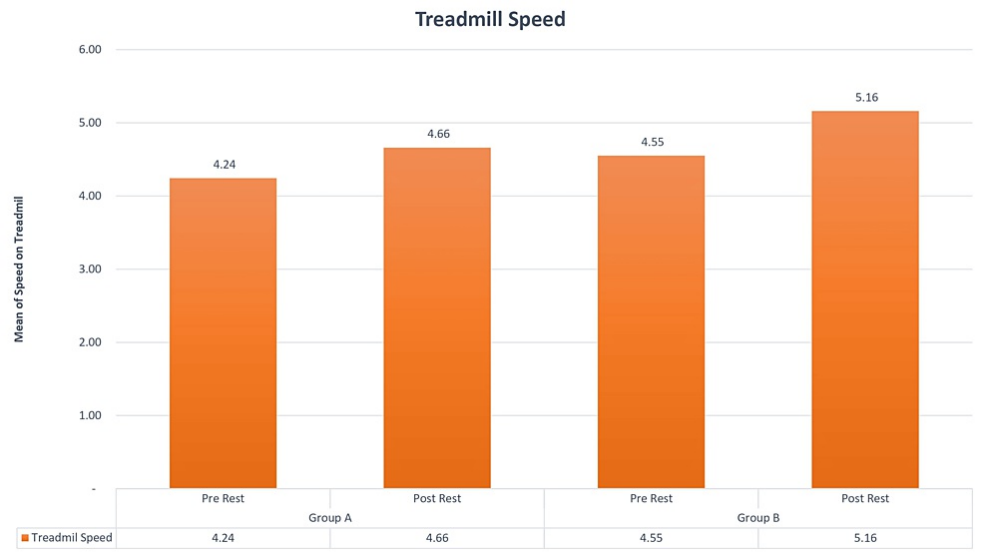
Comparisons of treadmill speed in Groups A and B in the pre and post test. The mean value of Group A in the pre test was 4.24, and in the post test, it was 4.66 with a 2% inclination. In Group B, the mean value of the pre test was 4.55, and the post-test value was 5.16 with a 2% inclination

**Figure 4:**
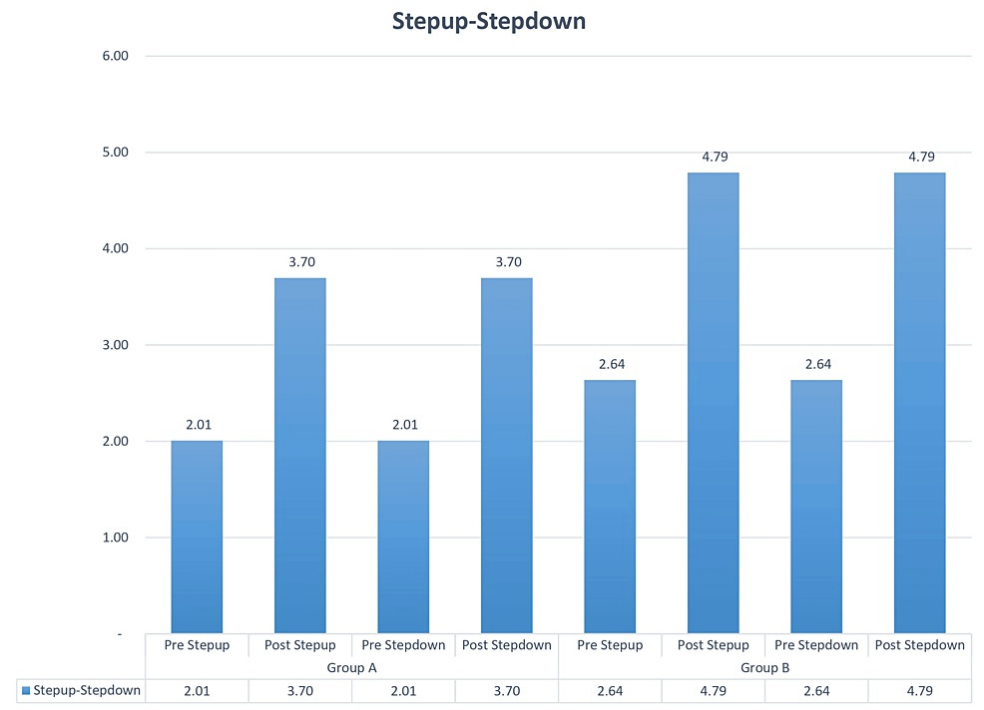
Comparisons of step-up and step-down tests in Groups A and B in the pre and post test. In the pre test, the mean value for Group A during the step-up test was 2.01, which subsequently increased to 3.70 in the post test. Similarly, during the step-down test, Group A recorded a mean value of 2.01 in the pre test and 3.70 in the post test. In contrast, Group B exhibited a mean value of 2.64 in the step-up pre test, which remarkably rose to 4.79 in the post test. Likewise, in the step-down pre test, Group B displayed a mean value of 2.64, which again increased to 4.79 in the post test

Based on gender, among the two groups, there were a total of 23 patients. In Group A, commonly called the Graston group, 12 were male, and 11 were female. Group B, the cupping group, included 11 male and 12 female participants. You can observe the results of the pre- and post-VAS test in Figure 1, while Figure 2 and Figure 3 display the findings from the pre- and post-treadmill time and speed tests. Figure 4 illustrates the outcomes of the pre- and post-step-up and step-down tests. Furthermore, Table 1 provides insights into the pre and post ROM, while Table 2 presents the pre- and post-MMT results.

**Table 1:** Comparisons of ankle range of motion in Groups A and B in the pre and post test, showing a significant effect with a value 0.04 S SD: standard deviation; SE: standard error; MD: mean difference; S: significant

			Mean	SD	SE	MD	T- value	p-value
Group A	Dorsiflexion	Pre test	13.96	1.74	0.36	4.09±1.62	2.11	0.04
		Post test	17.87	1.18	0.25			
	Plantarflexion	Pre test	30.96	2.20	0.46	5.04±1.80		
		Post test	36.39	1.08	0.22			
Group B	Dorsiflexion	Pre test	12.91	1.93	0.40	5.52±1.59		
		Post test	18.70	1.11	0.23			
	Plantarflexion	Pre test	32.83	2.67	0.56	6.00±2.30		
		Post test	38.43	1.44	0.30			

**Table 2:** Comparisons of manual muscle testing in Groups A and B in the pre and post test, showing a significant effect SD: standard deviation; SE: standard error

		Mean	SD	SE	T- value	p-value
Knee flexor	Group A	0.72	0.25	0.05	2.31	0.03
	Group B	0.54	0.26	0.05		
Knee extensor	Group A	0.74	0.56	0.12	-2.25	0.03
	Group B	1.07	0.41	0.08		
Dorsiflexion	Group A	0.59	0.33	0.07	-2.14	0.04
	Group B	0.78	0.29	0.06		
Plantarflexion	Group A	0.74	0.37	0.08	-2.23	0.03
	Group B	1.00	0.43	0.09		
Invertor	Group A	0.52	0.41	0.09	-2.47	0.02
	Group B	0.78	0.29	0.06		
Evertor	Group A	0.61	0.30	0.06	-2.11	0.04
	Group B	0.80	0.33	0.07		

## Discussion

Group A underwent the Graston technique, while Group B received dynamic cupping therapy. The treatment regimen extended over a three-week period, with patients receiving four sessions per week, totaling 12 sessions. Pain levels were assessed using a VAS, functional capabilities were gauged through step-up, step-down, and treadmill tests, the joint range was measured using a goniometer, and muscle strength was evaluated through MMT. Pain was monitored both before and after each treatment session. All assessment components were conducted before initiating treatment.

As pain diminished, improvements in functionality were observed and recorded through the treadmill test. The results indicated an enhanced duration of treadmill performance, especially with a 2% incline. Similar improvements were noted in the step-up and step-down tests. The tightness and soreness in specific muscles, including the tibialis anterior, tibialis posterior, soleus, peroneus longus, and peroneus brevis, negatively impacted the ROM. However, as muscle flexibility improved, positive effects on the ROM became evident.

The study suggests that the Graston technique primarily reduces pain and promotes healing by enhancing localized circulation. Increased surface temperature was observed after treatment, likely attributed to improved circulation and an influx of nutrients and fibroblasts, both of which aid in the healing process. Additionally, the Graston technique may help in breaking down scar tissue and reducing tissue adhesions. This study demonstrates that the Graston technique reduces subjective pain while enhancing the ROM [20].

A separate study examined the effects of the Graston technique and cupping therapy. It presented compelling evidence for the usefulness of both treatments in improving short- and long-term ROM. Cupping therapy, in particular, showed significant pain relief, supporting previous findings that it reduces subjective pain [21]. Adhesions in muscles can impair function, blood supply, and tissue nourishment, potentially leading to myofascial trigger points. Maximum temperature increases were observed approximately 25 minutes post treatment, suggesting that the therapeutic effects of the Graston technique/instrument-assisted soft tissue mobilization (IASTM) therapy persist beyond the session [22,23]. These findings align with the previous study, indicating that cupping treatment positively impacts muscular flexibility [24,25].

Cupping therapy is known to effectively manipulate physical structures such as fascia, skin, and musculocutaneous tissues. Muscle activation analysis revealed a significant increase, especially in the cupping therapy group, post intervention. This implies that cupping therapy alone leads to a considerable rise in muscle activation. The underlying theory suggests that cupping treatment aids in eliminating toxins and harmful elements from the treated area, contributing to its beneficial effects. Cupping creates negative pressure suction that helps draw out toxins, promoting granulation and wound healing. Multiple studies consistently support the notion that cupping is beneficial for early recovery in various conditions. Moreover, after undergoing cupping therapy, increased muscular activity and improved muscle flexibility were observed, potentially due to the interplay between muscle length and tension [26].

Comparing cupping treatment and the Graston technique/IASTM in terms of speed of results, both therapies showed a favorable effect on MTSS, with cupping therapy providing quicker pain relief and improved function due to its negative pressure mechanism. The application of cups in the affected area draws blood, activates ions, and stimulates the neuromuscular junction, reducing discomfort in three sessions and enhancing flexibility with ongoing therapy [27]. IASTM, while taking longer, yields similar results. Patients are advised to drink extra water after IASTM to prevent muscle discomfort and fatigue [16,24,27]. Both techniques proved beneficial for runners with MTSS, with cupping offering faster pain relief and the Graston technique delivering comparable results with the assistance of conventional methods.

The treadmill test not only serves as a valuable tool for studying human movement and exercise capacity but also plays a crucial role in training and conditioning. An interesting finding from this study is that high-level distance runners exhibit significantly better-running economy on a track compared to a treadmill, even with the commonly applied 2% incline setting [27]. Discrepancies between track and treadmill locomotion are primarily attributed to air resistance. In this study, pain intensity, as assessed with the VAS, influenced the timing of the treadmill test. When pain intensity decreased, treadmill test performance improved, correlating with increased performance in step-up and step-down tests. As strength and flexibility improved, so did the ROM. For more accurate results, a larger sample size is necessary, and long-term research is required to demonstrate its effectiveness.

## Conclusions

In this study, our primary focus was on individuals afflicted with MTSS who underwent a three-week treatment regimen involving the use of cupping therapy and the Graston technique, a form of IASTM. To assess the outcomes of these treatments, we employed various measures, including the VAS for pain assessment, treadmill test, step-up and step-down tests, ROM evaluation, and MMT. Upon comparing the results of the two treatment approaches, we observed notable variations. Both cupping therapy and the Graston technique exhibited positive effects and improvements in terms of ROM, pain alleviation, and functional enhancement. The key distinction between them lay in the time required for significant improvements to become apparent. It was evident that cupping therapy yielded faster and more pronounced effects compared to the Graston technique, especially in terms of the treatment duration. Subsequently, after conducting statistical analyses, we determined that cupping therapy offers greater benefits than the Graston technique for individuals grappling with MTSS, particularly in the realms of pain relief and functional improvement.
